# Das digitale Sportangebot „Get Up – Stand Up – Move Up“ während der Covid-19-Pandemie: eine Interviewstudie mit teilnehmenden Grundschulkindern

**DOI:** 10.1007/s43594-023-00089-w

**Published:** 2023-04-18

**Authors:** Julia Limmeroth, Lea Jebram, Florian Heussner, Norbert Hagemann, Volker Scheid

**Affiliations:** Institut für Sport und Sportwissenschaft, Universität Kassel, Damaschkestraße 25, 34121 Kassel, Deutschland

**Keywords:** Digitalisierung, Psychologische Grundbedürfnisse, Körperliche Aktivität, Freude, Qualitative Inhaltsanalyse, Covid-19, Digitalization, Basic psychological needs, Physical activity, Pleasure, Qualitative content analysis, Covid-19-pandemic

## Abstract

Die Covid-19-Pandemie führte während des zweiten Lockdowns zu einer Reduktion der körperlichen Aktivität der Kinder in Deutschland. Um den erschwerten Möglichkeiten, sich als Kind zu bewegen, etwas entgegenzusetzen, wurde ein digitales Sportangebot initiiert. Dieses nahm insbesondere die Befriedigung der psychologischen Grundbedürfnisse in den Blick und richtete sich an Kinder im Grundschulalter. Weiterhin stand die Vermittlung von Freude an der Bewegung im Fokus. Fünf bis sechs Wochen nach Projektstart wurden acht Interviews mit Kindern (*N* = 8) im Alter von 7 bis 10 Jahren (*M* = 8,38, *SD* = 1,19) geführt. Ein Mädchen, das ebenfalls am Projekt teilgenommen hatte, fungierte als Interviewerin. Die Interviews fanden digital via Zoom statt. Mittels einer qualitativen Inhaltsanalyse wurden die Ergebnisse zunächst kategorisiert und anschließend mit der Software MAXQDA Analytics analysiert. Grundsätzlich zeigte sich in den Aussagen der Kinder, dass insbesondere das Autonomie- und Kompetenzerleben befriedigt werden konnte. Hinsichtlich der sozialen Eingebundenheit ergab sich ein diverseres Bild, welches nur bedingt auf eine Förderung durch das digitale Format schließen lässt. Ungeachtet dessen berichteten alle Kinder davon, dass ihnen das digital vermittelte Sporttreiben Freude bereitet hätte. Abschließend werden mögliche Synergieeffekte hinsichtlich der Verknüpfung analoger und digitaler Formate diskutiert.

## Abstract (en)

Regarding the second lockdown during the Covid-19 pandemic, children in Germany were significantly less physically active. To counteract the more difficult opportunities to be physically active as a child, a digital sport- and exercise program was initiated. This approach focused on satisfying basic psychological needs and was aimed at children of primary school age. Furthermore, the focus lied on conveying the pleasure while exercising. Eight interviews (*N* = 8) were conducted with children aged 7–10 years (*M* = 8.38, *SD* = 1.19) five to six weeks after the start of the project. A girl, who also participated in the project, acted as the interviewer. The interviews were conducted digitally via Zoom. Using a qualitative content analysis, the results were first categorized and second analyzed using MAXQDA Analytics software. The children’s statements showed that especially the need for autonomy and competence could be satisfied. With regard to relatedness, the results were much more diverse. In particular, the support of relatedness was therefore only possible to a limited extent by means of this digital format. Notwithstanding this, all of the children reported that they had enjoyed to exercise also digitally. Finally, possible synergy effects with regard to linking analog and digital formats are discussed.

## Ausgangslage

Die Covid-19-Pandemie bestimmt seit Anfang 2020 das gesellschaftliche Leben und hat für viele Menschen einschneidende Veränderungen zur Folge: Viele Regierungen verhängten zur Eindämmung der rasch steigenden Infektionszahlen Lockdowns, deren Ziel es war, physische soziale Kontakte zu reduzieren, um dadurch die Infektionszahlen zu senken (Mutz und Gerke 2020). Dies hat auch für den (organisierten) Sport weitreichende Folgen: Sportvereine – ausgenommen der Profisport – waren teilweise gezwungen, ihr Sportangebot weitestgehend einzustellen beziehungsweise auf digitale Formate umzustellen (Kehl et al. 2021).

### Freizeitverhalten und körperliche Aktivität während der Lockdowns

Im Zusammenhang mit den Maßnahmen hat sich auch die Freizeitgestaltung der Heranwachsenden in Deutschland massiv verändert. Vor allem das Spielen zu Hause und dabei insbesondere die Beschäftigung mit digitalen Medien hat enorm zugenommen (Langmeyer et al. 2021), wobei schon vor der Pandemie die Reduzierung körperlicher Inaktivität über alle Generationen hinweg eine der größten Public-Health-Herausforderungen des 21. Jahrhunderts darstellte (z. B. Guthold et al. 2020).

**Figure 1:**
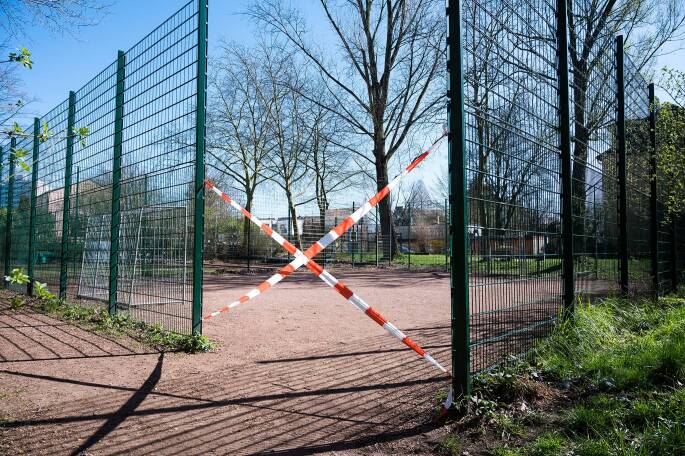

Mit Bezug zu den unterschiedlichen Lockdowns offenbaren repräsentative Daten aus Deutschland, dass diese differenziert voneinander zu betrachten sind: Im ersten Lockdown (Frühjahr 2020) zeigte sich, dass zwar die sportliche Betätigung der 4‑ bis 17-Jährigen zurückging (Abb. 1) und die Bildschirmzeit in der Freizeit zunahm, aber gleichzeitig eine deutliche Steigerung der gewohnheitsmäßigen körperlichen Aktivitäten (zum Beispiel Spazierengehen oder Spielen im Freien) zu verzeichnen war (Schmidt et al. 2020). Im zweiten Lockdown (Winter 2020/2021) zeigte sich für Deutschland jedoch ein Einbruch der körperlichen Aktivität auf Seiten der Kinder und Jugendlichen. Bedingt durch Faktoren wie der Jahreszeit, dem Wegfall des Schulsports und dem fehlenden Zugang zu Sportvereinen sank das Niveau unter das von vor der Pandemie (Schmidt et al. 2021). Außerdem nahm die Bildschirmzeit während der Pandemie zu. Kinder und Jugendliche waren gezwungen, Medien zu Kommunikationszwecken zu nutzen (Wong et al. 2021), wobei gleichzeitig alltägliche Wege zur Schule und in der Freizeit wegfielen. Der Großteil des Alltags verlagerte sich in den digitalen Raum (Bates et al. 2020).

### Digitalisierung und deren Auswirkungen

Für den Großteil der Heranwachsenden stellte das Digitale bereits vor der Covid-19-Pandemie einen alltäglichen Bestandteil ihres Lebens dar (Wiesemann et al. 2020). Wiederum haben die durch die Pandemie hervorgerufenen Einschränkungen und die damit einhergehende Reduktion der körperlichen Aktivität verschiedene Organisationen, unter anderem die Weltgesundheitsorganisation (2021) dazu veranlasst, die Notwendigkeit, zu Hause körperlich aktiv zu sein, hervorzuheben. Während der Pandemie hat sich auch das Angebot digitaler Kanäle, die verschiedene sportliche Aktivitäten anbieten, stark vergrößert (Nyenhuis et al. 2020). So sind vermehrt Kanäle entstanden, die auch die angeleitete Ausübung von körperlicher Aktivität unterstützen. Mittels Plattformen wie Youtube oder Zoom werden individualisierte Maßnahmen der körperlich-sportlichen Betätigung sowie Echtzeit-Kontakte digital präsentiert. Diese können unabhängig von ihrem Aufenthaltsort genutzt werden (Ng 2020). Solche Angebote richten sich vorwiegend an ältere Jugendliche bis hin zu Erwachsenen im mittleren Alter. Jüngere Kinder kamen oft erstmals durch die Pandemie in Kontakt mit solchen Vermittlungsangeboten (zum Beispiel „ALBAs tägliche Sportstunde“; Alba Berlin Basketballteam GmbH 2021). Ammar et al. (2020) schlagen vor, dass künftige Interventionen zur Förderung der körperlichen Aktivität in Zeiten einer Pandemie auf Informations- und Kommunikationstechnologien wie Fitness-Apps aufbauen könnten. Gleichzeitig ist jedoch festzustellen, dass der Digitalisierung im Kontext des Sports vor der Pandemie ein relativ geringer Stellenwert zugesprochen wurde (Morlang 2020). Daher könnten die Erfahrungen während der Pandemie genutzt werden, die Relevanz, die digitale Medien für junge Menschen besitzt (Wolfert und Pupeter 2018), aufzugreifen und zukünftig in die Gestaltung innovativer Sportangebote einzubeziehen.

### Körperlich-sportliche Aktivität und psychologische Grundbedürfnisse

Als theoretische Herangehensweise hat sich insbesondere die Selbstbestimmungstheorie (SDT) von Deci und Ryan (1985) in analogen, pädagogischen Settings als wesentliche Theorie etabliert. Ein zentraler Bestandteil der SDT stellen die psychologischen Grundbedürfnisse Autonomie, Kompetenz und soziale Eingebundenheit dar. Bezugnehmend auf Vansteenkiste, Ryan und Soenens (2020) bezieht sich Autonomie auf die Erfahrung der eigenen Willenskraft. Mit einem befriedigenden Autonomieerleben geht ein Gefühl der Integrität einher, insofern die eigenen Handlungen, Gedanken und Gefühle selbstbestimmt und authentisch sind. Kompetenz bezieht sich auf die Erfahrung von Wirksamkeit und erfolgreicher Bewältigung. Es stellt sich Zufriedenheit ein, wenn man in der Lage ist, sich an Aktivitäten zu beteiligen und Gelegenheiten wahrnimmt, seine Fähigkeiten und sein Fachwissen (Wissen?) einzusetzen/zu erweitern. Soziale Eingebundenheit meint die Erfahrung von Wärme, Bindung und Fürsorge. Sie wird befriedigt, wenn man sich mit anderen verbindet und sich für sie als wichtig empfindet.

Eine der Grundannahmen besteht darin, dass das soziale Umfeld beziehungsweise der soziale Kontext die eigene Wahrnehmung der Zufriedenheit mit der Befriedigung der psychologischen Grundbedürfnisse beeinflusst (Ryan und Deci 2000). Bei hinreichender Befriedigung, zum Beispiel durch ein entsprechendes Verhalten der Übungsleiter*innen, können damit unter anderem folgende positive Adaptionsprozesse einhergehen: ein höherer Grad an selbstbestimmter Motivation (Sylvester et al. 2018), positive Affekte und Freude (Álvarez et al. 2009), gesteigerter körperlicher Selbstwert (Sebire et al. 2009) und höhere Ausdauer und Anstrengungsbereitschaft (Ntoumanis 2005). Weiterhin schlussfolgern Guay et al. (2008) in ihrem Review, dass die von der SDT vorgeschlagenen Motivationstypen wichtig für das Verständnis davon sind, wie Schüler*innen „gedeihen“ und „erfolgreich“ sind. Die während der Covid-19-Pandemie durchgeführte Studie von Dana et al. (2021) stellt eine der wenigen Arbeiten dar, die im Rahmen eines Online-Sportunterrichts die SDT als theoretische Grundlage zur Konzeption einer digitalen, autonomiefördernden Interventionsmaßnahme genutzt haben. Insgesamt zeigte sich, dass die Interventionsgruppe (die autonomiefördernde Aufgaben erhielt) im Vergleich zur Kontrollgruppe eine höhere wahrgenommene Autonomieunterstützung, eine höhere Motivation und Intention zur körperlichen Aktivität sowie ein höheres körperliches Aktivitätsniveau im Vergleich von Pre- zu Posterhebung aufwies (im Pretest wiesen beide Gruppen ein ähnliches Niveau hinsichtlich der genannten Parameter auf). Gemäß Ntoumanis et al. (2021) wirken sich SDT-Interventionen positiv auf gesundheitsbezogene Korrelate aus. Die Effekte sind als mäßig und heterogen zu bezeichnen sowie zum Teil auf die Steigerung der selbstbestimmten Motivation und die Unterstützung durch soziale Akteur*innen zurückzuführen. Grundsätzlich jedoch hat sich die SDT in analogen Settings, insbesondere auch im Rahmen von Schulinterventionen, als konzeptioneller Rahmen für Sportinterventionsprogramme etabliert. So zeigen Vasconcellos et al. (2020) in ihrer Meta-Analyse zur SDT im Kontext des Schulsports, dass Sportlehrkräfte einen größeren Einfluss auf die Wahrnehmung von Autonomie und Kompetenz auf Seiten der Schüler*innen haben (wodurch adaptive Prozesse, wie zum Beispiel die Intention zum Sporttreiben, Selbstwirksamkeit etc. unterstützt werden können), während Gleichaltrige einen größeren Einfluss auf das Gefühl der sozialen Eingebundenheit aufweisen. Von den befriedigten Grundbedürfnissen korrelierte die Befriedigung der Kompetenz am stärksten mit der selbstbestimmten Motivation. Die Autor*innen schlussfolgern daraus, dass ein Gefühl der Wirksamkeit (der eigenen Kompetenz) im Sportunterricht besonders mit einer höheren Teilnahmebereitschaft im Sportunterricht verbunden ist.

### Die vorliegende Studie

Angesichts der Relevanzsteigerung, die mit der durch die Pandemie forcierten Digitalisierung des Sports einherging (Rode 2021), nutzt die vorliegende Studie einen qualitativen Ansatz, um mehr darüber zu erfahren, welche Aspekte zu einer positiven Beziehungsgestaltung auf digitaler Ebene im Kontext eines Sportangebots für Grundschulkinder führen können (Limmeroth et al. 2021). Dabei werden die Befriedigung der psychologischen Grundbedürfnisse und entsprechend angenommene Adaptionsprozesse in den Blick genommen. Weiterhin sollen erste Anknüpfungspunkte für Gelingensbedingungen digitaler, theoriegestützter Sportangebote ermittelt werden, die ein solches Angebot für Kinder attraktiv und im Sinne ihrer Entwicklung gestaltbar machen. Im Mittelpunkt der Studie stehen daher drei Fragestellungen:

- Wie erleben Kinder die Förderung von Autonomie, Kompetenz sowie sozialer Eingebundenheit innerhalb eines digitalen Sportangebots?
- Welche Faktoren begünstigen oder verhindern das Erleben von Freude und Spaß bei der Teilnahme an einem digitalen Sportangebot?
- Welche Besonderheiten kennzeichnen ein digitales Sportangebot im Unterschied zu analogen Settings?

## Methode

### Projektbeschreibung und Design

Mit „Get Up – Stand Up – Move Up“ wurde ein digitales Bewegungsprogramm entwickelt, das die angesprochenen Forderungen umsetzt und gemeinsam mit Kindern im Grundschulalter durchgeführt wurde. In der vorliegenden Studie wurden teilnehmende Kinder mittels qualitativer Verfahren zu ihrem Wohlbefinden im Projekt und zur Befriedigung ihrer psychologischen Grundbedürfnisse untersucht. Mithilfe der Online-Plattform Zoom wurden offene, teilstrukturierte Interviews durchgeführt und über dieses Medium aufgezeichnet. Die Aufnahmen wurden im Anschluss daran nach den vereinfachten Transkriptionsregeln von Kuckartz et al. (2007) transkribiert, wobei nur verbale Äußerungen übernommen wurden. Nonverbale Äußerungen wurden aufgrund des digitalen Formats nicht weiter berücksichtigt. Die Interviews wurden fünf bis sechs Wochen nach Projektstart (Mitte März 2021) geführt.

Für die Interviews wurde der methodische Ansatz „Kinder interviewen Kinder“ verfolgt (Trautmann 2010). Ein neun Jahre altes Mädchen hat die Rolle der interviewenden Person übernommen. Dieses Mädchen wurde ausgewählt, weil sie besonders kompetent erschien, die Interviews zu führen und selbst sehr motiviert war, als Interviewerin zu agieren. Der Kern des Ansatzes liegt darin, dass sich Kinder in einer ähnlichen Lebenswelt befinden und sich dadurch weniger Hemmungen auf Seiten der interviewten Kinder einstellen. Die Interviewerin war ebenfalls Teilnehmerin des Projekts und kannte daher die Abläufe aus eigener Erfahrung. Bei der Durchführung der Interviews war zu Beginn noch die Studienleitung in der Zoom-Sitzung anwesend, um den Ablauf der Interviews für alle Beteiligten zu erläutern. Danach verblieb die Studienleitung ohne Kamera und Mikro passiv in der Zoom-Sitzung.

An dieser Stelle soll kurz auf die generelle Besonderheit von Interviews mit Kindern und deren Analyse verwiesen werden: Kinder können sich, abhängig vom Alter, noch nicht in gleicher Weise wie Erwachsene artikulieren/ausdrücken. Sie verfügen aber bereits ab dem mittleren Grundschulalter über komplexe Denk- und Verhaltensmuster sowie die Fähigkeit zur Metakognition (Oerter 2008). Daher sieht zum Beispiel Heinzel (2012) den Erfolg der Kindheitsforschung daran gebunden, ob erwachsene Forscher*innen die Äußerungen von Kindern verstehen, entsprechend angemessen deuten und interpretieren können.

Das Projekt startete Anfang Februar 2021 mit dem Ziel, neben der Bewegungsförderung einen Schwerpunkt auf die Befriedigung der psychologischen Grundbedürfnisse zu legen (Deci und Ryan 1985) sowie die soziale Interaktion und die Beziehungsarbeit zu fokussieren. Über Videokonferenzen, die in einer Live-Session stattfanden, wurden die Bewegungseinheiten einmal wöchentlich für eine Stunde durchgeführt. Die acht Kleingruppen mit acht bis zehn Kindern wurden jeweils von zwei Personen angeleitet. Dabei wurden Tandems bestehend aus Jugendlichen des Vereins sowie Sportstudierenden gebildet. Inhaltlich wurde sich an den für diese Altersstufe zentralen Inhaltsfeldern wie zum Beispiel „Bewegen an und mit Geräten“ oder auch „Spielen“ orientiert (Hessisches Kultusministerium 2021): Die ersten Einheiten standen unter folgenden Mottos:

1. Laufen, Springen, Werfen: „Schnell wie ein Reh: Olympiade zu Hause“.
2. Turnerische Einheit mit einem Schwerpunkt auf der Förderung koordinativer und konditioneller Fähigkeiten: „Turnen wie ein Äffchen: Das eigene Zuhause ganz neu erkunden“.
3. Ästhetische und gestalterische Formen von Bewegung kennenlernen: „Bunt wie ein Chamäleon: Karneval des Sports zu Hause“.
4. Einsatzmöglichkeiten von Bällen und anderen Gegenständen in den eigenen vier Wänden: „Ballspielfreudig wie ein Delfin: Der Ball muss nicht immer ins Eckige“.

Eine detaillierte Projektbeschreibung findet sich bei Limmeroth et al. (2021). Konzeptionell wurde dem Verhalten der Übungsleitenden ein besonders hoher Stellenwert beigemessen. Ihr Verhalten und die Gestaltung des Sportangebots zielten darauf ab, Kompetenz- und Autonomieerleben auf Seiten der Kinder zu ermöglichen sowie soziale Eingebundenheit zu fördern.

### Stichprobe

Neun Kinder, die bereits vier bis fünf Wochen lang am Projekt teilgenommen hatten, meldeten sich auf Anfrage freiwillig zur Teilnahme an den Interviews. Ein Interview konnte leider nicht ausgewertet werden, da ein Elternteil wiederholt bei der Beantwortung der Fragen stellvertretend für das Kind gesprochen hatte. Alle anderen Interviews konnten ohne Kommentierung durch die Eltern beziehungsweise ohne deren Einmischung durchgeführt werden, sodass Interviews von acht Kindern (*N* = 8) im Alter von sieben bis zehn Jahren (*M* = 8,38, *SD* = 1,19) ausgewertet werden konnten. Ein Kind besuchte die 1. Klasse, zwei Kinder die 2. Klasse, ein Kind die 3. Klasse und vier Kinder die 4. Klasse. Sieben Kinder waren in einem Sportverein angemeldet.

### Untersuchungsmethoden

Es kamen offene, teilstrukturierte Interviews zum Einsatz, mit denen die drei forschungsleitenden Fragestellungen hinsichtlich der psychologischen Grundbedürfnisse, des Erlebens von Spaß und Freude sowie der Besonderheiten des digitalen Formats beantwortet werden sollten. Es wurde ein strukturierter Leitfaden für die Interviews gewählt. Der Leitfaden wurde in einer kindgerechten Sprache verfasst, um eine offene Beantwortung der Fragen zu gewährleisten. Im Leitfaden für die Kinderinterviews werden auf inhaltlicher Ebene Themenabschnitte abgearbeitet, die sich ähnlich wie die drei forschungsleitenden Fragestellungen im Wesentlichen auf verschiedene Aspekte der Befriedigung psychologischer Grundbedürfnisse, des Erlebens von Spaß und Freude sowie Aspekte des digitalen Formats beziehen. In Zusammenarbeit mit dem interviewenden Mädchen sowie mithilfe eines Vorinterviews wurde die kindgerechte Umsetzung des Leitfadens überprüft und entsprechend angepasst. Folgende exemplarische Fragen waren im Leitfaden enthalten:

- Kannst du mir ein Beispiel nennen, wo du in den Stunden auch mitbestimmen durftest oder sagen konntest, was du gerne machen würdest?
- Gab es bisher Aufgaben, die für dich zu leicht oder zu schwer waren?/Kannst du das genauer beschreiben?
- Welche Tipps und Hinweise geben dir (euch) die Übungsleitenden?
- Wie würdest du die Stimmung innerhalb der Gruppe beschreiben?
- Worauf freust du dich, wenn du an die nächste digitale Sportstunde denkst?
- Was würdest du dir für das Projekt und für das digitale Sporttreiben wünschen?

### Qualitative Datenanalyse

Mit der qualitativen Inhaltsanalyse wurde ein Analyseverfahren gewählt, welches Kommunikationsinhalte mit dem Ziel einer stark regelgeleiteten Interpretation dieser Inhalte systematisiert (Stamann et al. 2016). Im Zentrum steht ein theoriegeleitetes und am Material entstandenes Kategoriensystem, dessen Vorteil in der möglichen Quantifizierung von kodierten Textstellen auf Basis einer regelgeleiteten Zuordnung zu den Kategorien liegt (Mayring 2020). Als Grundlage für die Inhaltsanalyse wurden die Transkripte der aufgezeichneten Interviews verwendet. Ausgehend von theoretischen Überlegungen wurden Kategorien gebildet, die im Wesentlichen dem konzeptionellen Ansatz des Sportprogramms entsprechen. Zur Analyse und Systematisierung der Daten wurde die Software MAXQDA Analytics Pro 2022 (Release 22.0.0) eingesetzt.

Zunächst wurde ein erstes übergeordnetes Kategoriensystem entwickelt und auf die Transkripte angewendet. Dabei wurden in einem ersten Schritt die Kategorien Autonomiebedürfnis, Kompetenzerleben und soziale Eingebundenheit sowie die Kategorien Spaß/Freude am Programm und digitales Format aufgestellt. In einem zweiten Schritt wurden ergänzende Subkategorien gebildet, die eine weitere Differenzierung der Inhalte ermöglichten. Das Kategoriensystem ist in Tab. 1 dargestellt.

**Table 1:** 

Kategorie	Subkategorie	Definition	Quelle	Ankerbeispiel	Kodierregeln
**Autonomie**	*Mitbestimmung*	Demokratische Entscheidungsfindung (Mitbestimmung und Auswahloptionen)	Amorose & Anderson-Butcher (2007)	„Ich kann mir manchmal Sachen wünschen, die wir dann manchmal machen“ (B4, Abs. 41)	(fehlende) Möglichkeiten der Einbringung/Wahloptionen und Berücksichtigung der Einbringung durch ÜL ^a^
	*Entscheidung*	Aufnahme, Beibehalten und Regulation von Verhalten	Miserandino (1996)	„Dann haben wir überlegt, ob wir da mitmachen wollen“ (B5, Abs. 25)	Entstehung von eigenen Entscheidungen sowie Einflussnahmen auf diese
**Kompetenz**	*Aufgabenvielfalt*	Aspekt des „mastery climates“ Kompetenzerleben und Erfolgserlebnisse	Valentini & Rudisill (2004); Li et al. (2005)	„Also mir fehlt sehr viel, dass man nur ganz wenige Sachen machen kann“ (B2, Abs. 49)	Abwechslung bei den Übungen und Fehlen von Aufgaben oder Möglichkeiten für Handlungen
	*Hilfestellung*	Positives, ermutigendes und hilfreiches Feedback Instruktionen	Allen & Howe (1998)	„Deswegen unterstützen sie auch (…) und (…) helfen halt auch immer“ (B5, Abs. 55)	Unterstützung der Kinder bei der Ausführung von Aufgaben oder Problemen mit der eigenen Kompetenz
	*Aufgabenschwierigkeit*	Angemessene Schwierigkeit und Differenzierung	Li et al. (2007)	„Die Aufgaben (…) sind jetzt nicht so schwer“ (B3, Abs. 33)	(Nicht‑) Angemessenheit von Aufgabenschwierigkeit sowie Über- bzw. Unterforderung
**Soziale Eingebundenheit**	*Beziehung zu Übungsleitenden*	Positive Wahrnehmung der Übungsleitenden (Stundengestaltung)	Riley & Smith (2011)	„Mir gefällt an ihnen, dass sie halt auch sehr nett sind und nicht zu streng“ (B1, Abs. 57)	Beziehung zwischen den Übungsleiter*innen und den Kindern
	*Beziehung zu Kindern*	Sozialer Kontakt zu anderen Kindern, Kennenlernen und Freundschaften	Pacewicz et al. (2020)	„Ich kenn die anderen Kinder jetzt nicht so (…) und ich kenn auch manchmal nicht mehr ihre Namen“ (B3, Abs. 71)	Beziehungen zwischen einzelnen Kindern (u. a. soziale Kontakte oder Interaktionen)
	*Gruppengefühl*	Stimmung, Zugehörigkeit zur Gruppe, Rituale	Erikstad et al. (2018)	„Bei uns wird eigentlich niemand ausgeschlossen in der Gruppe“ (B5, Abs. 75)	Die Gruppe als betrachtete Einheit; Gruppenzugehörigkeit und interne Zustände
	*Gemeinsamer Sport*	Zusammenarbeit, Gesellschaft anderer beim Sport	Rackow et al. (2014)	„(…) dass ich andere (…) wiedersehe, mit denen ich Sport machen kann“ (B6, Abs. 31)	Gemeinsame sportliche Aktivität und Aspekte der gemeinsamen Aufgabenbewältigung
**Spaß und Freude**	*Bedingungen*	Aspekte, die das Erleben von Spaß und Freude fördern	Dismore & Bailey (2011)	„Mir macht es Spaß und es sind auch lustige Übungen“ (B5, Abs. 27)	Bedingungen, unter denen Spaß/Freude empfunden wurden
	*Feedback*	Positives Feedback zum Programm drückt Spaß/Freude aus	Lorusso et al. (2013)	„Also mir gefällt (…) es bis jetzt sehr gut. Mir macht das auch Spaß“ (B3, Abs. 27)	Formen der affektiven Rückmeldung bezogen auf das Programm/die Gestaltung
	*Motivation*	Spaß/Freude zeigt sich in Form von Motivation zum Sporttreiben	Schneider & Kwan (2013)	„(…) dass es noch ein bisschen länger geht“ (B4, Abs. 51)	Motivation zur Teilnahem an den Stunden und dem Programm insgesamt
**Digitales Format**	*Eindruck*	Wahrnehmung des digitalen Formats	–	„Also den Eindruck finde ich da sehr gut“ (B2, Abs. 45)	Rückmeldungen zum Programm mit Bezug zum digitalen Format (ohne *Einschränkungen*)
	*Unterschiede*	Vergleich zu Schul‑, Vereins-, und Freizeitsport	–	„Viele andere Übungen als wie wir die in der Schule machen“ (B7, Abs. 67)	Konkret vergleichende Erwähnungen von digitalen und anderen (analogen) Formaten
	*Einschränkungen*	Grenzen und Problematiken des digitalen Formats	–	„Ich kann jetzt nicht über den Bildschirm den Ball zuwerfen“ (B3, Abs. 51)	Einschränkungen und Probleme im Rahmen der digitalen Durchführung des Programms

^a^ *ÜL* Übungsleitende

## Ergebnisse

Die Ergebnisse der Kinderinterviews sind zusammenfassend in Tab. 2 dargestellt und werden im Folgenden entsprechend der Leitfragen sowie in Anlehnung an die theoretische Konzeption der SDT beschrieben:

**Table 2:** 

Kategorie	Subkategorie	Kernergebnisse	Nennungen
**Autonomie**	*Mitbestimmung*	Wahloptionen und eigene Gestaltungsmöglichkeiten	(7)
		Wünsche wurden von ÜL^a^ erfragt und berücksichtigt	(4)
		Sich einbringen, nur möglich bei ausreichend Zeit	(1)
	*Entscheidung*	Teilnahme bei allen durch Eltern angeregt	(8)
		(Eigene) Entscheidung nicht erkennbar	(5)
		Eigene Entscheidung für Teilnahme	(2)
		Eltern fordern von Kind, einen Sport auszuüben	(1)
**Kompetenz**	*Aufgabenvielfalt*	Fehlende Möglichkeiten	(5)
		Vielfalt im/am Programm	(2)
		Wunsch nach bekannten Aktivitäten	(2)
	*Hilfestellung*	ÜL^a^ korrigierten & modifizierten Ausführung	(8)
		Kinder fühlten sich unterstützt	(7)
		Kinder fragten bei eigenen Problemen bei ÜL^a^ nach	(7)
	*Aufgabenschwierigkeit*	Ausgewogene Schwierigkeit	(9)
		Keine Überforderung	(6)
**Soziale Eingebundenheit**	*Beziehung zu Übungsleitenden*	ÜL^a^ waren „nett“, „cool“ (…)	(4)
		ÜL^a^ gestalteten Aufgaben lustig	(2)
		Kinder erinnerten teils nicht die Namen der ÜL^a^	(2)
	*Beziehung zu Kindern*	Kinder kannten einige Kinder teilweise vorher	(7)
		Keine neuen Freundschaften wurden geschlossen	(6)
		Kennenlernen war möglich und fand statt	(4)
		Keine Unterhaltungsmöglichkeiten	(1)
	*Gruppengefühl*	Kinder fühlten sich alle als Teil der Gruppe	(8)
		Ritual wird genannt	(6)
		Niemand wird aus der Gruppe ausgeschlossen	(1)
		Durch neu dazukommende Kinder wird es „komisch“	(1)
	*Gemeinsamer Sport*	Kinder wünschten sich, mehr gemeinsam zu machen	(7)
		Kinder wurden von ÜL^a^ ermutigt, gemeinsam zu arbeiten	(5)
		Kinder freuten sich auf gemeinsames Sporttreiben	(3)
**Spaß und Freude**	*Bedingungen*	Gesellschaft der anderen Kinder	(3)
		Abwechslung bei den Stundeninhalten	(2)
		Körperliche Anstrengung	(2)
		Lustige Aufgabengestaltung	(2)
		Spezifische Aktivitäten	(2)
	*Feedback*	Positives Feedback: Kinder fanden Programm gut	(17)
		Ein Kind langweilte sich etwas in einer Stunde	(1)
	*Motivation*	Große Mehrheit ist (hoch) motiviert für nächste Einheit	(8)
		Das Programm soll auch „nach Corona“ weitergehen	(2)
**Digitales** **Format**	*Eindruck*	Das Konzept gefällt gut	(2)
		Es macht auch Spaß und ist cool	(2)
		Es ist nicht schlechter als andere Formate	(1)
	*Unterschiede*	In Schule und Verein ist es „mehr zusammen“	(5)
		Es sind andere Übungsformen	(4)
		Man hat andere Möglichkeiten z. B. in einer Halle	(4)
		Es gibt kaum Unterschiede	(2)
	*Einschränkungen*	Technische Probleme (z. B. schlechte Internetverbindung)	(5)
		Weniger Möglichkeiten (z. B.durch fehlende Geräte)	(4)
		Man sieht sich nicht richtig	(3)
		Sozialer Kontakt fehlt/ist schwieriger	(3)
		Fehlende Formate (z. B. Teamsport)	(2)

*Hinweis.* Aufgrund von inhaltlichen als auch konzeptionellen Überschneidungen einiger Kategorien wurden. Doppelkodierungen akzeptiert

^a^ *ÜL* Übungsleitende

### Psychologische Grundbedürfnisse

#### Autonomie.

Im Hinblick auf das Autonomieerleben durften die Kinder mitbestimmen und ihre eigenen Ideen einbringen („Ich kann mir manchmal Sachen wünschen, die wir dann manchmal machen“, B4, Abs. 41). Es wurde ihnen ermöglicht, bei der Gestaltung des Programms und dessen Durchführung an wiederholter Stelle mitzuentscheiden. Die Übungsleitenden haben die Wünsche der Kinder erfragt und berücksichtigt. Es wurden Optionen zur Wahl gestellt, Vorschläge der Kinder aufgenommen sowie Übungs- und Spielformen durch Ideen der Kinder erweitert. Ein Mangel an Befriedigung der Autonomie wurde nur sehr vereinzelt angegeben. Zum Beispiel merkte ein Kind an, dass die Mitbestimmung nur „am Ende, wenn wir dann noch Zeit haben“, möglich war. Wiederum spielten bei der Entscheidung für die Teilnahme am Programm bei allen Kindern die Eltern eine tragende Rolle, aber nur einem Kind wurde „gesagt, [es müsse] irgendwo am Sport teilnehmen“ und deswegen „habe [es] eben bei dem Onlinetraining mitgemacht“ (B8, Abs. 27).

#### Kompetenz.

Alle Kinder fühlten sich von den Übungsleitenden bei den Übungen unterstützt und gaben an, dass diese mit Feedback und Tipps geholfen hätten. Ein Kind äußerte zur Hilfestellung, „dass die [Übungsleiter*innen] auch wollen, dass wir [Kinder] es richtig machen und deswegen unterstützen sie [uns] auch“ (B5, Abs. 39). Weiterhin geht aus den Kinderinterviews übereinstimmend hervor, dass die Übungen angemessen gestaltet waren und es zu keiner Überforderung gekommen ist. Die Aufgabenschwierigkeit wurde durch eine Mischung aus leichten und schweren Aufgaben im Schnitt als „meistens genau richtig“ (B1, Abs. 35) beschrieben. Zur Aufgabenvielfalt äußerte ein Kind: „(…) dass wir auch ganz unterschiedliche Dinge machen, finde ich eigentlich ganz gut“ (B4, Abs. 65), wobei Aufgabenvielfalt hier im Zusammenhang der Differenzierungsmöglichkeiten angesehen wird. Ausbleibende Befriedigung der Kompetenz wurde von den Kindern, ähnlich wie beim Autonomieerleben, nur vereinzelt wiedergegeben. Das Kompetenzerleben wurde vor allem durch die begrenzten Möglichkeiten, Aufgaben vielfältig zu gestalten, eingeschränkt. Die Kinder deuteten hier vermehrt an, „dass man nur ganz wenige Sachen machen kann und jetzt nicht an Geräten und so“ (B2, Abs. 49).

#### Soziale Eingebundenheit.

Beim Grundbedürfnis der sozialen Eingebundenheit ließen sich sowohl einige positive als auch mehrere negative Aspekte hinsichtlich der Bedürfnisbefriedigung feststellen. Überwiegend positiv waren die Aussagen der Kinder zum Gruppengefühl und zur Beziehung zu den Übungsleiter*innen. Es „[wurde] eigentlich niemand ausgeschlossen in der Gruppe“ (B5, Abs. 75) und „es [war] immer ganz lustig“ (B3, Abs. 67). Den Kindern gefiel an den Übungsleiter*innen, „dass sie halt auch sehr nett [waren] und nicht zu streng“ (B1, Abs. 57), „alles gut erklären“ (B2, Abs. 53) und „alles so lustig rüberbringen“ (B5, Abs. 53). Negative Aspekte ergaben sich dahingehend, dass zum Beispiel das Hinzukommen neuer Kinder „komisch“ war (B4, Abs. 77) und diese sich oft nicht an die Namen ihrer Übungsleiter*innen erinnern konnten. Darüber hinaus fallen die Äußerungen zum gemeinsamen Sporttreiben und den Beziehungen zu anderen Kindern heterogener aus. Die Kinder wurden zwar angeregt, zusammen mit den anderen Kindern zu arbeiten, gaben aber gleichzeitig an, dass sie es „lieber [mögen], wenn man zusammen ist“ (B1, Abs. 47). Zusätzlich störte es sie, „dass [man] nicht alle anderen sehen [konnte]“ (B5, Abs. 49). Allerdings gaben die Kinder zum Teil auch an, dass sie sich bei den Stunden explizit darauf freuen würden, Sport gemeinsam mit anderen beziehungsweise mit ihren Freund*innen treiben zu können. Ungeachtet dessen „hat man sich so [auch] ein bisschen kennengelernt“ (B1, Abs. 43) und vereinzelt konnten die Kinder neue Freundschaften schließen. Gleichzeitig äußerten viele Kinder, dass „[Freundschaften zu schließen] ja jetzt über digital nicht so gut [geht]“ (B3, Abs. 79) und sie sich vor allem mit den Kindern unterhalten haben, die sie bereits vor dem Programm schon kannten und „sonst [mit] keine[n] neuen“ (B6, Abs. 115). Insgesamt gaben alle Kinder an, sich als Teil einer Gruppe zu fühlen und schätzten die Stimmung innerhalb ihrer Gruppe als gut ein. Außerdem berichteten sie von Ritualen, die sie als Gruppe durchführten. Auch das gemeinsame Ritual zum Bewegungsablauf „Get Up – Stand Up – Move Up“ wurde explizit genannt.

### Spaß und Freude am Programm

Das Feedback zum Programm fiel überwiegend positiv aus. Die Kinder nannten unter anderem die Abwechslung bei den Übungen, die Gesellschaft der anderen Kinder, die körperliche Anstrengung, die lustige Stundengestaltung sowie jeweils ganz konkrete Aktivitäten beziehungsweise Spiele als Gründe, warum ihnen die Einheiten Spaß bereitet hätten. Außerdem waren sie nach eigener Einschätzung im Durchschnitt hoch motiviert für die nächsten Sitzungen und freuten sich darauf. Weiterhin gaben die Kinder an, sich konkret „auf den Spaß mit den anderen Kindern“ (B2, Abs. 31) zu freuen, der sie in der nächsten Stunde erwarten würde. Einmal wurde geäußert, dass die zurückliegende Stunde „ein bisschen langweilig“ (B4, Abs. 35) war. Insgesamt brachten alle Kinder positive Rückmeldungen zum Programm zum Ausdruck. Bei der Frage, was sie selbst, wenn sie einmal Übungsleiter*in sein könnten, anders machen würden, wurde angegeben, „auch viel Musik“ einzubauen und die Stunden „länger [zu] machen“ (B4, Abs. 73), aber auch gar nichts ändern zu wollen, weil eigentlich „macht es […] Spaß, so wie es ist“ (B5, Abs. 53).

### Digitales Format

Bezogen auf das digitale Format war der Eindruck der Kinder „sehr gut“ (B2, Abs. 45) und „auch nicht schlechter“ (B4, Abs. 55) als andere (analoge) Formate: „so (…) [geht es] halt auch“ (B1, Abs. 47). Gleichzeitig äußerten die Kinder deutlich, dass „es natürlich noch besser [wäre], würden [sie sich] nicht digital treffen“ (B5, Abs. 81) und sie es lieber mögen, „wenn man zusammen ist und es zusammen macht“ (B1, Abs. 47). Bezugnehmend auf diese Äußerungen offenbarten sich zwei zentrale Aspekte: 1. Es hat auch in einem digitalen Setting funktioniert, (gemeinsam) Sport zu treiben. 2. Die Kinder würden in einem direkten Vergleich zum größten Teil ein analoges Setting vorziehen, da sie in diesem eher das Gefühl hätten, wirklich gemeinsam Sport zu treiben. Neben der Einschränkung des sozialen Miteinanders gaben die Kinder vor allem an, dass „man ja jetzt nicht joggen [kann] zum Beispiel“ (B6, Abs. 59) oder „nicht über den Bildschirm den Ball zuwerfen [kann]“ (B3, Abs. 51). Außerdem wurde geäußert, dass „die Internetverbindung bei manchen nicht so gut [sei]“ (B3, Abs. 45). Einen Unterschied zu anderen Formaten sahen einige Kinder „eigentlich gar keinen“ (B2, Abs. 65) und andere gaben vor allem an, „dass es immer andere Übungen sind“ (B5, Abs. 67) bzw. „viele andere Übungen als die, [die wir] in der Schule machen“ (B7, Abs. 67). Darüber hinaus wurde der Wunsch geäußert, „dass das [Programm] auch noch weitergeht, wenn das mit Corona alles vorbei ist“ (B4, Abs. 91).

## Diskussion

Zunächst ist anzumerken, dass mit dieser qualitativen Studie vielfach „Neuland“ betreten wurde. Zum einen wurden Interviews digital, mittels des Videokonferenz-Tools Zoom, geführt und zusätzlich wurde ein Setting gewählt, in dem ein Kind ein anderes Kind interviewt. Zum anderen beziehen sich die Interviews auf die Umsetzung eines Sportangebots für Kinder im digitalen Raum, dessen sportwissenschaftliche Betrachtung bislang noch kaum erfolgt ist (Rode 2021).

### Befriedigung psychologischer Grundbedürfnisse und Spaß am Programm

Primär war es Ziel dieser Studie, zu untersuchen, ob die psychologischen Grundbedürfnisse – Autonomie, Kompetenz und soziale Eingebundenheit – innerhalb eines digitalen Sportangebots für Kinder gefördert werden können. Zusätzlich wurde in diesem Zusammenhang betrachtet, ob das digitale Sportangebot Spaß bereitet und welche Möglichkeiten beziehungsweise Grenzen sich durch das Format ergeben.

#### Autonomie.

Grundsätzlich zeigt sich in den Aussagen der Kinder, dass aus ihrer Perspektive die Übungsleitenden viel Wert darauf gelegt haben, Autonomie zu fördern. Die Kinder führen an, dass sie sehr unterschiedliche Übungen, Spielformen etc. gemacht haben. Sie konnten sich einbringen und die Übungsleiter*innen haben sich bemüht, auf ihre Wünsche einzugehen. Insgesamt geht aus den Aussagen hervor, dass es entsprechend zu einer Befriedigung dieses Bedürfnisses auf Seiten der teilnehmenden Kinder gekommen ist. Wie aus unterschiedlichen Interventionsstudien, die in analogen Settings durchgeführt wurden, hervorgeht (vgl. zum Beispiel das Review von Leisterer und Jekauc (2020)), trägt die Förderung des Erlebens von Autonomie dazu bei, die Motivation zur Aktivität selbst zu steigern. Zudem bestehen auch langfristige positive Wirkungen auf eine allgemein erhöhte Motivation, sportlich aktiv zu sein. Der letztgenannte Aspekt geht zum Beispiel aus dem Review zu SDT-Interventionsstudien im Gesundheitsbereich von Ntoumanis et al. (2021) hervor, wonach die Zunahme der autonomen Motivation und des Bedürfnisses nach Unterstützung am Ende der Intervention jeweils mit positiven Veränderungen des Gesundheitsverhaltens und der psychischen Gesundheit am Ende der Intervention verbunden sind. Allerdings ergaben sich in der vorliegenden Studie durch fehlende Möglichkeiten innerhalb des digitalen Settings auch Einschränkungen hinsichtlich der Befriedigung des Autonomiebedürfnisses. Beispielsweise stellte es eine Herausforderung dar, eine Aufgabenvielfalt innerhalb der Stunden zu verankern, die auch (individuelle) Wahlmöglichkeiten (von mehreren Kindern) beinhalteten. Ungeachtet dessen deuten die Äußerungen der Kinder mehrheitlich darauf hin, dass die „Umstände“ ihr Autonomieerleben nicht negativ beeinflusst haben, sondern eher im Gegenteil auch im digitalen Raum Autonomie erlebt werden konnte (Tilga et al. 2021a).

#### Kompetenz.

Hinsichtlich des Kompetenzerlebens legen die Aussagen der Kinder nahe, dass die Übungsleitenden immer wieder versucht haben, die Aufgabenschwierigkeit zu differenzieren und Feedback bei der Ausführung zu geben. So wurde die Aufgabenschwierigkeit als „meistens genau richtig“ eingeschätzt. Beispielhaft kann für das Bemühen der Übungsleiter*innen und ihre Unterstützung die Aussage angeführt werden, „dass die [Übungsleiter*innen] auch wollen, dass wir [Kinder] es richtig machen und deswegen unterstützen sie [uns] auch“ (B5, Abs. 39). Als einschränkend für das digitale Format lässt sich aus den Aussagen der Kinder ableiten, dass individuelle Stärken nicht immer gezeigt werden können beziehungsweise ihnen nicht immer ausreichend Beachtung geschenkt werden kann. Allerdings deutet sich auch für das Kompetenzerleben an, dass dieses grundsätzlich gefördert und im Rahmen der Möglichkeiten des digitalen Sportprogramms befriedigt werden konnte. Bezugnehmend auf die Meta-Analyse von Sheeran et al. (2020) hinsichtlich SDT-basierten Interventionen zur Änderung des Gesundheitsverhaltens zeigt sich, dass neben der bereits angesprochenen autonomen Motivation die wahrgenommene Kompetenz Gesundheitsverhalten vorhersagen kann. Diese beiden Variablen sind demnach entscheidend für die Auswirkungen von SDT-Interventionen auf das Gesundheitsverhalten. Wobei davon auszugehen ist, dass autonome Motivation doch nach der SDT aus wahrgenommener Kompetenz folgt, also potenziell vermittelt wird. Entsprechende Bemühungen – dass die Kinder sich als kompetent erleben – könnten sich also auch langfristig positiv auswirken.

#### Soziale Eingebundenheit.

Hinsichtlich der sozialen Eingebundenheit wird deutlich, dass das digitale Format die Möglichkeiten zur Förderung einschränkt und dies mit einer geringeren Befriedigung der sozialen Eingebundenheit auf Seiten der Kinder einhergeht. Neben diesen eher negativen, einschränkenden Aspekten zeigt sich jedoch in den Aussagen der Kinder ebenso, dass vor allem die Beziehung zu den Übungsleitenden sowie die Anwesenheit anderer Kinder von Relevanz war, um in abgeschwächter Weise soziale Eingebundenheit in einer Gruppe zu erleben. Daher könnte über digitale Formate zumindest eine Art initiale Grundlage geschaffen werden, um Beziehungen zu Gleichaltrigen aufzubauen beziehungsweise aufrechtzuerhalten. In Zeiten der Pandemie war dies sicherlich von besonderer Relevanz. In diesem Zusammenhang wurde von den Kindern auch die Bedeutung des Anfangs- und Abschlussrituals (siehe Limmeroth et al. 2021, S. 62) vielfach hervorgehoben, wodurch sie sich als Teil einer Gruppe wahrnehmen konnten. Insgesamt offenbart sich bei der Durchführung vor allem ein großer Nachteil: eine erschwerte Interaktion bedingt durch das digitale Format. Die erschwerte Kommunikation zu den anderen Kindern und damit ein geringeres Erleben sozialer Eingebundenheit steht wiederum in Einklang mit den Ergebnissen von Vasconcellos et al. (2020), die zeigen konnten, dass insbesondere Gleichaltrige das Gefühl der sozialen Eingebundenheit beeinflussen. Gleichzeitig sollte in diesem Zusammenhang hervorgehoben werden, dass es für die heutige Lebenswelt der Kinder etwas vollkommen „Normales“ darstellt, „dass Menschen mit Menschen interagieren, die nicht da sind, d. h. nicht körperlich analog anwesend sind“ (Wiesemann et al. 2020, S. 6). Insofern digitale Medien analoge Interaktionen nicht ersetzen, sondern ergänzen, könnten sie daher durchaus einen Mehrwert auch in Hinblick auf das Erleben sozialer Eingebundenheit darstellen.

#### Spaß am Programm: das digitale Format im Spiegel der psychologischen Grundbedürfnisse.

Insgesamt geben die Kinder an, Spaß an dem Programm gehabt und sich jeweils auf die nächste Stunde gefreut zu haben. In Einklang mit Sebire et al. (2013) und Álvarez et al. (2009) liegt es daher nahe, dass Interventionen zur Steigerung der körperlichen Aktivität so konzipiert sein sollten, dass Kinder Spaß an der Bewegung haben und eine inhärente Befriedigung in der Bewegung verspüren. Spaß und Freude an der Bewegung – das heißt positive affektive Erfahrungen mit körperlicher Aktivität – korrelieren stark positiv mit dem entsprechenden körperlichen Aktivitätsverhalten (Moore et al. 2009). Das Erleben positiver Affekte könnte dadurch zu einem nachhaltigeren körperlichen Aktivitätsverhalten über die gesamte Lebensspanne hinweg führen (Ekkekakis und Brand 2021). Grundsätzlich lässt sich hinsichtlich der Befriedigung der psychologischen Grundbedürfnisse bezugnehmend auf die betrachtete Intervention ein positives Fazit ziehen. In Anlehnung an die SDT (Ryan und Deci 2000) ist davon auszugehen, dass eine entsprechende Gestaltung eines Sportprogramms, die Wert auf ein positives Klima, Mitbestimmung und Eigenständigkeit legt, mit positiven Effekten hinsichtlich der Befriedigung psychologischer Grundbedürfnisse einhergeht (Standage et al. 2012). Dies wiederum kann zu einer generell gesteigerten Motivation zum Sporttreiben, zu mehr Freude am Sport und zu einem gesteigerten Wohlbefinden führen (Teixeira et al. 2012). Aus den Aussagen der Kinder lässt sich ableiten, dass der Fokus auf die Befriedigung der psychologischen Grundbedürfnisse eine gute Herangehensweise für die Konzeption digitaler Sportangebote sein kann. So greifen unter anderem Sebire et al. (2013) einen der Kerngedanken der SDT auf, dass die Befriedigung der psychologischen Grundbedürfnisse ein möglicher Weg zu autonomer Sportmotivation bei Kindern darstellt und soziale Akteur*innen, wie in der vorliegenden Studie die Übungsleitenden, durch zum Beispiel das Anwenden entsprechender zwischenmenschlicher Kommunikationsstrategien zu dieser Befriedigung entscheidend beitragen können (Sebire et al. 2016; Standage und Ryan 2012).

Die größte Schwierigkeit, und damit auch eine Begrenztheit des digitalen Formats, scheint in der Befriedigung der sozialen Eingebundenheit zu liegen, während Kompetenz- und Autonomieerleben relativ erfolgreich gefördert beziehungsweise befriedigt wurden. Bezugnehmend auf die Meta-Analyse von Sheeran et al. (2020) bestimmen wesentlich das Erleben von Autonomie und Kompetenz den Erfolg SDT-basierter Interventionen. Dies steht ebenso in Einklang mit Erkenntnissen von Vasconcellos et al. (2020), wonach die Befriedigung des Kompetenzerlebens am stärksten mit dem Grad selbstbestimmter Motivation korrelierte. Nichtsdestotrotz sollte die Bedeutung sozialer Eingebundenheit nicht unterschätzt werden, da insbesondere das Erleben sozialer Eingebundenheit bei weniger sportinteressierten Personen einen möglichen kompensatorischen Weg darstellt, über den sich autonome Motivation einstellen kann (Cox und Williams 2008). Innerhalb der dargelegten Studie stellte sich die Befriedigung sozialer Eingebundenheit über einen digitalen Weg als schwierig dar. Demzufolge ist die Bedeutung eines gemeinsamen, analogen Sportreibens hervorzuheben.

### Limitationen

Im Hinblick auf die Limitationen der Studie muss auf die Art der Befragung und den Umfang der Stichprobe eingegangen werden. Auch wenn die Methodik „Kinder interviewen Kinder“ als grundsätzlich positiv und innovativ einzuordnen ist, gab es durchaus auch einige Schwierigkeiten: das interviewende Kind konnte nicht so gut Gesprächspausen aushalten und drängte daher häufiger auf Antworten der befragten Kinder. Zusätzlich war der digitale Raum als Befragungsort komplett neu und stellte für die Kinder, neben der ungewohnten Interviewsituation, eine weitere, zusätzliche Herausforderung dar.

Kritisch anzumerken ist weiterhin, dass acht Kinderinterviews nur einen ersten Einblick in die Umsetzung und Wirkung des Projekts geben können und sich Anknüpfungspunkte für zukünftige Studien bieten. Um die Befunde abzusichern und allgemeine Empfehlungen zur Gestaltung digitaler Sportangebote für Kinder ableiten zu können, müssten weitere empirische Untersuchungen erfolgen, die sowohl auf qualitative als auch quantitative Herangehensweisen (unter anderem mit größeren Stichproben) setzen.

### Ausblick: Synergien sichtbar und nutzbar machen

**Figure 2:**
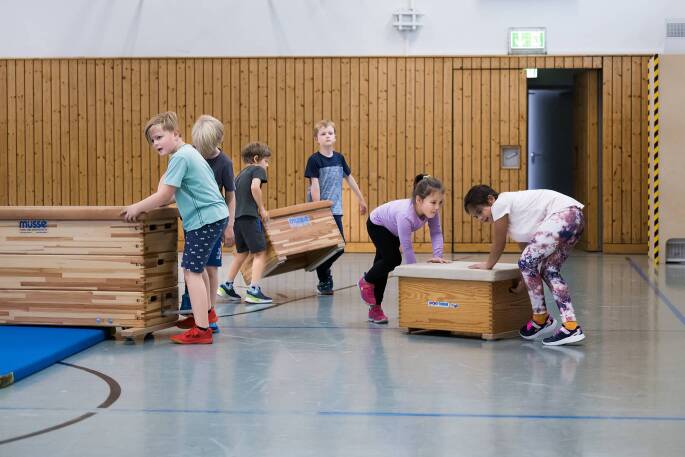

Auch wenn dem digitalen Sporttreiben Grenzen gesetzt sind, so gibt es auch Chancen, die beispielsweise in der Ergänzung analoger Angebote oder der Implementierung hybrider Formate liegen könnten (Tilga et al. 2021b). Im Hinblick auf das durchgeführte Sportangebot zeigt sich beispielsweise in dem Wunsch, dass das Angebot auch nach Corona weitergehen solle, dass auch digitale Angebote Kinder erfolgreich für Bewegung und Sport begeistern können. Zusätzlich legen die Ergebnisse der World Vision-Studie aus dem Jahre 2018 nahe, dass der digitale Raum für Kinder bereits zu einem immanenten Bestandteil ihres Lebens geworden ist. So ist die Nutzung des Internets unter der Woche im Jahre 2018 im Vergleich zur Erhebung von 2013 von 18 auf 38 % bei den 6‑ bis 11-Jährigen angestiegen (Wolfert und Pupeter 2018). Bei den 10- bis 11-Jährigen gaben 2018 sogar 67 % an, regelmäßig unter der Woche das Internet zu nutzen. Davon ausgehend könnte konkreter an der Umsetzung digitaler Formate, losgelöst von einem Vergleich mit analogen Settings (Amann 2020), gearbeitet werden. Hierbei könnten Überlegungen eine Rolle spielen, auf welche Weise diese für welche Zielgruppe eingesetzt oder zu hybriden Angebotsformen ausgestaltet werden könnten. Die Ergebnisse der vorliegenden Studie sollten als Anknüpfungspunkt für Interventionsansätze zu digitalen Sportangeboten basierend auf den psychologischen Grundbedürfnissen verstanden werden. Aus den Ergebnissen lässt sich schlussfolgern, dass vor allem das Autonomie- und das Kompetenzerleben auch innerhalb eines digitalen Sportangebots gefördert sowie befriedigt werden können, was einen Beitrag zu einer generellen Sportmotivation leistet (vgl. Dana et al. 2021). Zeigten sich vor und auch zu Beginn der Pandemie noch keinerlei Zusammenhänge zwischen der Bildschirmzeit und dem körperlichen Aktivitätsverhalten bei Kindern (Schmidt et al. 2020), so weisen die Ergebnisse von Wunsch et al. (2021) nun jedoch darauf hin, dass die Bildschirmzeit vor Covid-19 die körperliche Aktivität während Covid-19 negativ beeinflusst hat. Umso wichtiger erscheint es, die negativen Auswirkungen der Covid-19-Pandemie in den Blick zu nehmen und gleichzeitig positive Synergieeffekte aufzudecken. Zum einen könnten durch digitale Formate Bewegungs- und Sportangebote für bestimmte Zielgruppen attraktiver werden. Zum anderen könnten in hybriden Formaten neue Potenziale für das Erlernen von Bewegungen und das Begleiten von Trainingsmaßnahmen erschlossen werden, die einer fortschreitenden Digitalisierung Rechnung tragen (Wiesemann et al. 2020). Aus den genannten Gründen sollten digitale beziehungsweise hybride Formate für einzelne Zielgruppen weiterentwickelt und besser untersucht werden, um davon ausgehend empirisch gestützte Empfehlungen für den Schul- und Vereinssport ableiten zu können (Rode 2021). Gleichzeitig ist zu betonen, dass dem analogen Sporttreiben, insbesondere im Kindes- und Jugendalter, eine enorme Relevanz hinsichtlich der Möglichkeit sozialer Begegnungen und des sozialen Miteinanders zukommt (Abb. 2) und dies mittels digitaler Medien nicht ersetzt werden kann.

## References

[CR1] Alba Berlin Basketballteam GmbH Reaktion auf Coronavirus: ALBAs tägliche digitale Sportstunde für Kinder und Jugendliche. https://www.albaberlin.de/news/details/reaktionauf-coronavirus-albas-taegliche-digitalesportstunde-fuer-kinder-und-jugendliche/ (Zugegriffen: 12. Dez. 2021).

[CR55] Allen JB, Howe BL (1998). Player ability, coach feedback, and female adolescent athletes’ perceived competence and satisfaction. Journal of Sport and Exercise Psychology.

[CR2] Álvarez MS, Balaguer I, Castillo I, Duda JL (2009). Coach autonomy support and quality of sport engagement in young soccer players. Spanish Journal of Psychology.

[CR3] Amann K, Wiesemann J, Eisenmann C, Fürtig I, Mohn BE (2020). Kindheit unter Beobachtung. Digitale Kindheiten.

[CR4] Ammar A, Brach M, Trabelsi K, Chtourou H, Boukhris O, Masmoudi L, Bouaziz B, Bentlage E, How D, Ahmed M, Muller P, Muller N, Aloui A, Hammouda O, Paineiras-Domingos LL, Braakman-Jansen A, Wrede C, Bastoni S, Pernambuco CS, Hoekelmann A (2020). Effects of COVID-19 home confinement on eating behaviour and physical activity: Results of the ECLB-COVID19 international online survey. Nutrients.

[CR51] Amorose AJ, Anderson-Butcher D (2007). Autonomy-supportive coaching and self-determined motivation in high school and college athletes: A test of self-determination theory. Psychology of Sport and Exercise.

[CR5] Bates LC, Zieff G, Stanford K, Moore JB, Kerr ZY, Hanson ED, Barone Gibbs B, Kline CE, Stoner L (2020). COVID-19 impact on behaviors across the 24-hour day in children and adolescents: Physical activity, sedentary behavior, and sleep. Children.

[CR6] Cox A, Williams L (2008). The roles of perceived teacher support, motivational climate, and psychological need satisfaction in students‘ physical education motivation. Journal of Sport and Exercise Psychology.

[CR7] Dana A, Khajeaflaton S, Salehian MH, Sarvari S (2021). Effects of an intervention in online physical education classes on motivation, intention, and physical activity of adolescents during the COVID-19 pandemic. International Journal of School Health.

[CR8] Deci EL, Ryan RM (1985). Intrinsic motivation and selfdetermination in human behavior.

[CR61] Dismore H, Bailey R (2011). Fun and enjoyment in physical education: Young people’s attitudes. Research Papers in. Education.

[CR9] Ekkekakis P, Brand R, Englert C, Taylor I (2021). Exercise motivation from a post-cognitivist perspective: affective-reflective theory. Motivation and selfregulation in sport and exercise.

[CR59] Erikstad MK, Martin LJ, Haugen T, Høigaard R (2018). Group cohesion, needs satisfaction, and self-regulated learning: A one-year prospective study of elite youth soccer players’ perceptions of their club team. Psychology of Sport and Exercise.

[CR10] Guay F, Ratelle CF, Chanal J (2008). Optimal learning in optimal contexts: The role of selfdetermination in education. Canadian Psychology.

[CR11] Guthold R, Stevens GA, Riley LM, Bull FC (2020). Global trends in insufficient physical activity among adolescents: a pooled analysis of 298 population-based surveys with 1·6 million participants. The Lancet Child and Adolescent Health.

[CR12] Heinzel F, Heinzel F (2012). Qualitative Methoden in der Kindheitsforschung. Ein Überblick. Methoden der Kindsheitsforschung. Ein Überblick über Forschungszugänge zur kindlichen Perspektive.

[CR13] Hessisches Kultusministerium (2021). Bildungsstandards und Inhaltsfelder: Das neue Kerncurriculum für Hessen. Primarstufe. Sport. https://kultusministerium.hessen.de/sites/kultusministerium.hessen.de/files/2021-06/kerncurriculum_primarstufe_sport.pdf. Zugegriffen: 1. November 2021

[CR14] Kehl M, Strobl H, Tittlbach S, Loss J (2021). „Der Mensch, der Handball spielt, braucht den Ball, den Kontakt und die Gemeinschaft“ – Veränderungen im Sportangebot durch die COVID-19 Pandemie und deren Bedeutung fur Sportvereine. Gesundheitswesen.

[CR15] Kuckartz U, Dresing T, Rädiker S, Stefer C (2007). Qualitative Evaluation in sieben Schritten.

[CR16] Langmeyer A, Naab T, Winklhofer U, Guglhör-Rudan A, Urlen M, Dohmen D, Hurrelmann K (2021). Kind sein in Zeiten von Corona. Generation Corona? Wie Jugendliche durch die Pandemie benachteiligt werden.

[CR17] Leisterer S, Jekauc D (2020). Influencing adolescents’ affective states in physical education via autonomy-supportive teaching styles: A systematic review of intervention studies. International Journal of Physical Education.

[CR54] Li W, Lee AM, Solmon MA (2005). Relationships among dispositional ability conceptions, intrinsic motivation, perceived competence, experience, and performance. Journal of Teaching in Physical Education.

[CR56] Li W, Lee A, Solmon M (2007). The role of perceptions of task difficulty in relation to self-perceptions of ability, intrinsic value, attainment value, and performance. European Physical Education Review.

[CR18] Limmeroth J, Hagemann N, Heussner F, Scheid V (2021). Gelingensbedingungen eines digitalen Sportangebots. Forum Kinder- und Jugendsport.

[CR62] Lorusso JR, Pavlovich SM, Lu C (2013). Developing student enjoyment in physical education. Physical and. Health Education Journal.

[CR19] Mayring P, Mey G, Mruck K (2020). Qualitative Inhaltsanalyse. Handbuch Qualitative Forschung in der Psychologie.

[CR52] Miserandino M (1996). Children who do well in school: Individual differences in perceived competence and autonomy in above-average children. Journal of Educational Psychology.

[CR20] Moore JB, Yin Z, Hanes J, Duda JL (2009). Measuring enjoyment of physical activity in children: Validation of the physical activity enjoyment scale. Journal of Applied Sport Psychology.

[CR21] Morlang K (2020). Digitalisierung. Forum Kinder- und Jugendsport.

[CR22] Mutz M, Gerke M (2020). Sport and exercise in times of self-quarantine: How Germans changed their behaviour at the beginning of the Covid-19 pandemic. International Review for the Sociology of Sport.

[CR23] Ng K (2020). Adapted physical activity through COVID-19. European Journal of Adapted Physical Activity.

[CR24] Ntoumanis N (2005). A prospective study of participation in optional school physical education using a self-determination theory framework. Journal of Educational Psychology.

[CR25] Ntoumanis N, Ng JYY, Prestwich A, Quested E, Hancox JE, Thogersen-Ntoumani C, Deci EL, Ryan RM, Lonsdale C, Williams GC (2021). A meta-analysis of self-determination theory-informed intervention studies in the health domain: effects on motivation, health behavior, physical, and psychological health. Health Psychology Review.

[CR26] Nyenhuis SM, Greiwe J, Zeiger JS, Nanda A, Cooke A (2020). Exercise and fitness in the age of social distancing during the COVID-19 pandemic. The Journal of Allergy and Clinical Immunology: In Practice.

[CR27] Oerter R, Oerter R, Montada L (2008). Kindheit. Entwicklungspsychologie.

[CR58] Pacewicz CE, Smith AL, Raedeke TD (2020). Group cohesion and relatedness as predictors of self-determined motivation and burnout in adolescent female athletes. Psychology of Sport and Exercise.

[CR60] Rackow P, Scholz U, Hornung R (2014). Effects of a new sports companion on received social support and physical exercise: An intervention study. Applied Psychology: Health and Well. Being.

[CR57] Riley A, Smith AL (2011). Perceived coach-athlete and peer relationships of young athletes and self-determined motivation for sport. International Journal of Sport Psychology.

[CR28] Rode D, Steinberg C, Bonn B (2021). Digitalisierung als kultureller Prozess – Grundlegende Bestimmungen und sportpädagogische Anschlüsse jenseits der Technologie. Digitalisierung.

[CR29] Ryan RM, Deci EL (2000). Self-determination theory and the facilitation of intrinsic motivation, social development, and well-being. American Psychologist.

[CR30] Schmidt SCE, Anedda B, Burchartz A, Eichsteller A, Kolb S, Nigg C, Niessner C, Oriwol D, Worth A, Woll A (2020). Physical activity and screen time of children and adolescents before and during the COVID-19 lockdown in Germany: a natural experiment. Scientific Reports.

[CR63] Schneider ML, Kwan BM (2013). Psychological need satisfaction, intrinsic motivation and affective response to exercise in adolescents. Psychology of Sport and Exercise.

[CR31] Schmidt SCE, Burchartz A, Kolb S, Niessner C, Oriwol D, Hanssen-Doose A, Worth A, Woll A (2021). Zur Situation der körperlich-sportlichen Aktivität von Kindern und Jugendlichen während der COVID-19 Pandemie in Deutschland: Die Motorik-Modul Studie (MoMo).

[CR34] Sebire SJ, Standage M, Vansteenkiste M (2009). Examining intrinsic versus extrinsic exercise goals: cognitive, affective, and behavioral outcomes. Journal of Sport and Exercise Psychology.

[CR33] Sebire SJ, Russell J, Fox KR, Edwards MJ, Thompson JL (2013). Testing a self-determination theory model of children’s physical activity motivation: a cross-sectional study. International Journal of Behavioral Nutrition and Physical Activity.

[CR32] Sebire SJ, Kesten JM, Edwards MJ, May T, Banfield K, Tomkinson K, Blair PS, Bird EL, Powell JE, Jago R (2016). Using self-determination theory to promote adolescent girls’ physical activity: Exploring the theoretical fidelity of the Bristol Girls Dance Project. Psychology of Sport and Exercise.

[CR35] Sheeran P, Wright CE, Avishai A, Villegas ME, Lindemans JW, Klein WMP, Rothman AJ, Miles E, Ntoumanis N (2020). Self-determination theory interventions for health behavior change: Meta-analysis and meta-analytic structural equation modeling of randomized controlled trials. Journal of Consulting and Clinical Psychology.

[CR36] Stamann C, Janssen M, Schreier M (2016). Qualitative Inhaltsanalyse – Versuch einer Begriffsbestimmung und Systematisierung. Forum Qualitative Sozialforschung.

[CR38] Standage M, Ryan RM, Roberts GC, Treasure DC (2012). Self-determination theory and exercise motivation: facilitating self-regulatory processes to support and maintain health and well-being. Advances in motivation in sport and exercise.

[CR37] Standage M, Gillison FB, Ntoumanis N, Treasure DC (2012). Predicting students’ physical activity and health-related well-being: a prospective cross-domain investigation of motivation across school physical education and exercise settings. Journal of Sport and Exercise Psychology.

[CR39] Sylvester BD, Curran T, Standage M, Sabiston CM, Beauchamp MR (2018). Predicting exercise motivation and exercise behavior: A moderated mediation model testing the interaction between perceived exercise variety and basic psychological needs satisfaction. Psychology of Sport and Exercise.

[CR40] Teixeira PJ, Carraça EV, Markland D, Silva MN, Ryan RM (2012). Exercise, physical activity, and self-determination theory: A systematic review. International Journal of Behavioral Nutrition and Physical Activity.

[CR41] Tilga H, Kalajas-Tilga H, Hein V, Koka A (2021). Web-based and face-to-face autonomy-supportive intervention for physical education teachers and students’ experiences. Journal of Sports Science and Medicine.

[CR42] Tilga H, Kalajas-Tilga H, Hein V, Raudsepp L, Koka A (2021). Effects of a web-based autonomy-supportive intervention on physical education teacher outcomes. Education Sciences.

[CR43] Trautmann T (2010). Interviews mit Kindern. Grundlagen, Techniken, Besonderheiten, Beispiele.

[CR53] Valentini NC, Rudisill ME (2004). Motivational climate, motor-skill development, and perceived competence: Two studies of developmentally delayed kindergarten children. Journal of Teaching in Physical Education.

[CR44] Vansteenkiste M, Ryan RM, Soenens B (2020). Basic psychological need theory: Advancements, critical themes, and future directions. Motivation and Emotion.

[CR45] Vasconcellos D, Parker PD, Hilland T, Cinelli R, Owen KB, Kapsal N, Lee J, Antczak D, Ntoumanis N, Ryan RM, Lonsdale C (2020). Self-determination theory applied to physical education: A systematic review and meta-analysis. Journal of Educational Psychology.

[CR46] Wiesemann J, Eisenmann C, Fürtig I, Lange J, Mohn BE, Wiesemann J, Eisenmann C, Fürtig I, Lange J, Mohn BE (2020). Digitale Kindheiten. Kinder – Familien – Medien. Digitale Kindheiten.

[CR47] Wolfert S, Pupeter M, Andresen S, Neumann S (2018). Freizeit: Hobbys und Mediennutzung. Kinder in Deutschland 2018. 4. World Vision Kinderstudie.

[CR48] Wong CW, Tsai A, Jonas JB, Ohno-Matsui K, Chen J, Ang M, Ting DSW (2021). Digital screen time during the COVID-19 pandemic: Risk for a further myopia boom?. American Journal of Ophthalmology.

[CR50] World Health Organization. (2021). #HealthyAtHome—Physical Activity. https://www.who.int/news-room/campaigns/connecting-the-world-to-combat-coronavirus/healthyathome/healthyathome---physical-activity. Zugegriffen: 10. November 2021.

[CR49] Wunsch K, Nigg C, Niessner C, Schmidt SCE, Oriwol D, Hanssen-Doose A, Burchartz A, Eichsteller A, Kolb S, Worth A, Woll A (2021). The impact of COVID-19 on the interrelation of physical activity, screen time and health-related quality of life in children and adolescents in Germany: results of the Motorik-Modul study. Children.

